# An unusual presentation of adult T-cell leukemia/lymphoma

**DOI:** 10.3332/ecancer.2018.801

**Published:** 2018-01-23

**Authors:** Mohammed Tag-Adeen, Keiichi Hashiguchi, Yuko Akazawa, Ken Ohnita, Sawayama Yasushi, Niino Daisuke, Kazuhiko Nakao

**Affiliations:** 1 Department of Gastroenterology and Hepatology, Nagasaki University Hospital, 1-7-1 Sakamoto, 8528501 Nagasaki-shi, Nagasaki, Japan; 2 Department of Internal Medicine, Qena School of Medicine, South Valley University, Qena 83523, Egypt; 3 Department of Haematology, Nagasaki University Hospital, 1-7-1 Sakamoto, 8528501 Nagasaki-shi, Nagasaki, Japan; 4 Department of Pathology, Nagasaki University Hospital, 1-7-1 Sakamoto, 8528501 Nagasaki-shi, Nagasaki, Japan

**Keywords:** adult T-Cell leukaemia/lymphoma, human T-lymphotropic virus, oropharyngeal ulcers, oral ulcers, endoscopic findings, lymphoma

## Abstract

Adult T-cell Leukemia/Lymphoma (ATL) is a rare disease, related to human T-lymphotropic virus-1 (HTLV-1) and presented mainly in adulthood by generalised lymphadenopathy, hepatosplenomegaly, skin lesions and hypercalcaemia, with rare gastrointestinal and/or oral manifestations. We reported this case to raise awareness and demonstrate the therapeutic challenges of this rare disease. A 49-year-old Japanese female presented with skin papules on both forearms, painful mouth ulcers and multiple neck swellings since early February 2017. Initial clinical examination and laboratory investigations were misleading and her condition was diagnosed as candidiasis. Because of un-improvement of the case, a screening upper endoscopy was requested 2 months later and revealed characteristic oropharyngeal ulcers which were biopsied, and its pathologic examination confirmed smouldering type ATL. This case report should raise awareness of doctors and endoscopists about this disease especially in HTLV-1 endemic areas to avoid late diagnosis and help achieve earlier therapeutic decisions.

## Introduction

Adult T-cell Leukemia/Lymphoma (ATL) is a specific variant of peripheral T-cell lymphoma that mainly presents in endemic areas of Japan and recognises the human T-lymphotropic virus-1 (HTLV-1) as an etiological agent [1].

The natural history, clinical characteristics and prognosis of ATL are greatly variable and serve as the basis for classification of the disease into five clinical types: smouldering, chronic, primary cutaneous tumoral, lymphoma and acute. The smouldering type is subdivided into leukaemic and non-leukaemic, and the chronic type into favorable and unfavorable [2, 3].

Gastrointestinal involvement occurs secondary to systemic disease in about one third of ATL cases with the stomach being involved in 40%, small intestine in 38% and large intestine in 34% [1, 4]. However, no endoscopic pattern could be related to a peculiar ATL subtype, and smoldering and chronic subtypes do not typically show GI involvement [1, 5].

Clinically ATL diagnosis should be based on seropositivity for HTLV-1 performed by enzyme-linked immunesorbent assay and confirmed by Western blot and/or polymerase chain reaction (PCR) in association with haematological and/or histopathological diagnoses of peripheral T-cell leukemia/lymphoma [2, 6]. Flow cytometry is an important test for ATL diagnosis, the minimum markers required for it should include CD3, CD4, CD7, CD8, CD25 and Ki-67 while most ATL patients display a mature CD4 cells phenotype [2, 7].

Patients with tissue infiltration should undergo biopsy and pathological examination to investigate the type of viral integration in the peripheral blood mononuclear cells and/or fresh neoplastic tissue [3, 6]; detection of monoclonal infiltrate by either reverse and long-range PCR [8] or Southern blot [9] confirms ATL diagnosis.

The aim of this case report was to identify a rare presentation of smoldering type ATL which was detected during endoscopic screening and confirmed by histopathologic examination.

## Case report

### Initial presentation

A female patient, 49 years old, from Nagasaki, Japan, presented since early February 2017 with skin papules on both forearms, then she developed a painful mouth ulcer with multiple neck swellings by late February. She sought the medical advice of her dentist who recommended referral to internal medicine department for hospitalisation.

Initial examination at the internal medicine department revealed a painful small ulcer at the middle of the soft palate with a pain score of 5/18 and cervical lymph node swellings. The complete blood count was normal and a biopsy showed no malignancy. The condition was diagnosed as candidiasis, and anti-fungal treatment was prescribed with an improvement of the pain score of 1/18 but healing of the ulcer was not noticed despite adherence to therapy.

### Laboratory investigations

By late March 2017, progression of the palatal ulcer and non-improvement of cervical lymphadenopathy indicated re-admission of the patient for further clinical assessment and laboratory investigations. A full blood count (Table 1) showed normal red blood cells (RBCs) count and morphology (RBCs count: 4.4 × 10^6^, normal: 3.8–4.9 × 10^6^) and normal platelets count and morphology (platelets count: 274 × 10^3^, normal: 158–348 × 10^3^). The total leukocytic count was normal: 8.3 × 10^3^ (3.3–8.6 × 10^3^) but 40% of leucocytes were abnormal lymphocytes (abnormal lymphocytic count: 3.3 × 10^3^, normal: 0). Biochemical and immunological investigations (Table 2) revealed normal lactate dehydrogenase: 172 U/L (124–222 U/L), normal serum calcium (8.8-10.1 mg/dL), and negative antinuclear antibody. High level of HTLV-1 monoclonal bands was recognised by Southern blot method (>45, normal: <1 cutoff-index), also serum level of soluble interleukin-2 receptor (sIL-2R) was very high (3420 U/mL) which then raised to 5960 U/mL one month later (in asymptomatic HTLV-1 carrier it should not exceed 800 U/mL). Flow cytometry showed CD4: 77.9%, CD8: 12.3%, CD25: 72.1%, CCR4: 68.1% and TSLC1: 68.7%.

### Endoscopic screening

**Figure 1.**
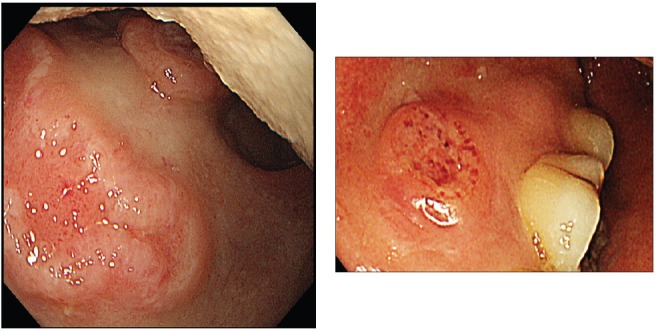
Endoscopic picture of ulcers at the soft palate and gingiva with irregular margins, raised edges and erythematous floors.

**Figure 2.**
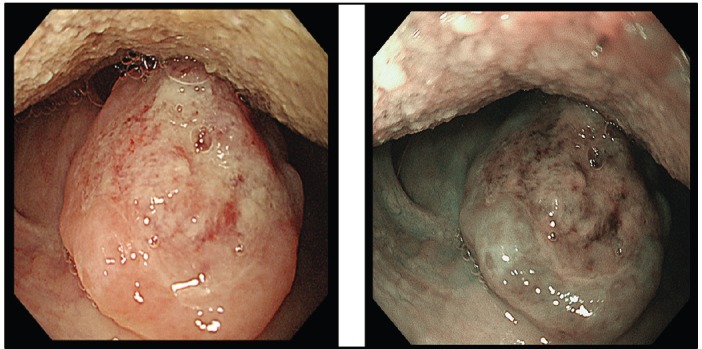
Ulcer at the uvula with white light endoscopy (left) and NBI (right).

**Figure 3.**
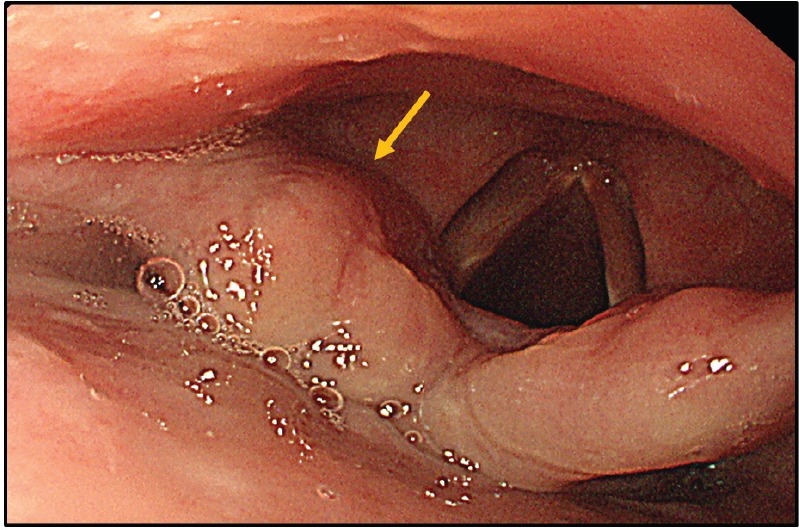
Thickened aryepiglottic fold (yellow arrow) suspicious of infiltration.

**Figure 4.**
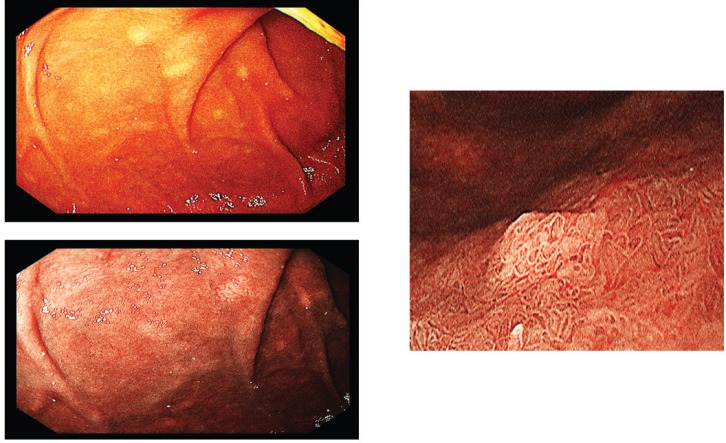
Multiple submucosal swellings at the second part of the duodenum as seen by white light endoscopy (upper left), NBI (lower left) and magnified view (right).

A screening upper endoscopy with narrow band imaging (NBI) showed multiple oral and pharyngeal ulcers with irregular margins, raised edges and erythematous floors (Figures 1 and 2), thick aryepiglottic folds (Figure 3), multiple small gastric fundic gland polyps and multiple small submucosal duodenal swellings (Figure 4). Biopsies were taken from the pharyngeal and duodenal lesions for tissue diagnosis.

### Imaging findings

Head and neck computerised tomography (CT) showed mild bilateral cervical and axillary lymphadenopathy with left maxillary sinus effusion. Chest CT: ground glass infiltration of the right lung upper lobe apical segment mostly inflammatory and multiple sub-pleural lung nodules about 6 mm in diameter scattered in the right lung lower lobe and left lung upper lobe with thickening of the adjacent pleura. Abdominal CT: mild free ascites and gall bladder wall thickening mostly adenomyomatosis. Liver, spleen and other abdominal organs were normal.

Whole body positron emission tomography-CT (PET-CT) revealed dense accumulation of fluorodeoxyglucose (FDG) in the pharynx, palate and tip of the tongue, lymph nodes affection including axillary, cervical, para-aortic, porta hepatis, iliac and inguinal lymph nodes, parenchymal lung nodular sub-pleural infiltration, and skin involvement including scalp, back of the neck, both upper arms, both forearms, first finger of the left hand and fifth finger of the right hand.

### Tissue diagnosis

**Figure 5.**
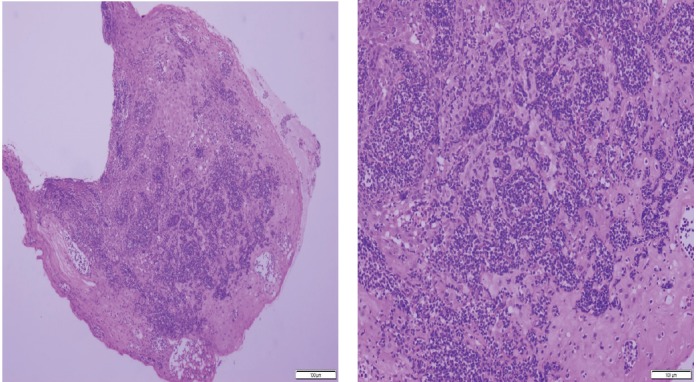
Histopathologic sections from oropharyngeal mucosa, stained by hematoxylin & eosin with different magnification powers (×4: left side, ×8: right side); showing malignant lymphocytic infiltration with hyperchromatic enlarged nuclei and scanty cytoplasm.

**Figure 6.**
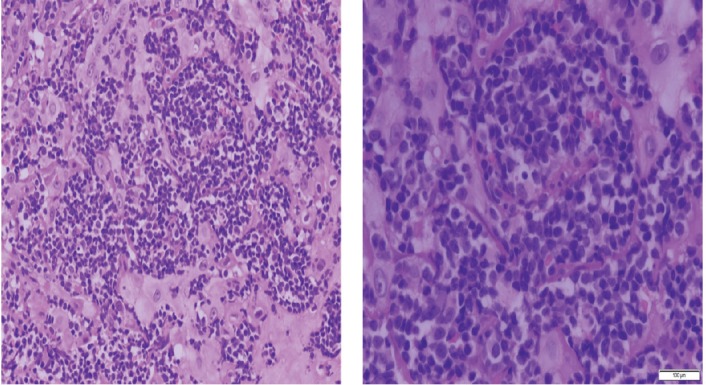
Histopathologic sections stained by hematoxylin & eosin from the same specimen with higher magnification powers (×20: left side, ×40: right side).

**Figure 7.**
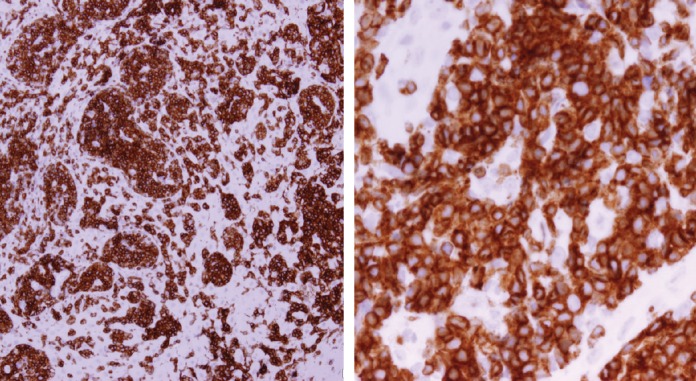
Immune-histochemical staining of the same sections showing CD3-positive cells (×20: left side, ×40: right side).

**Figure 8.**
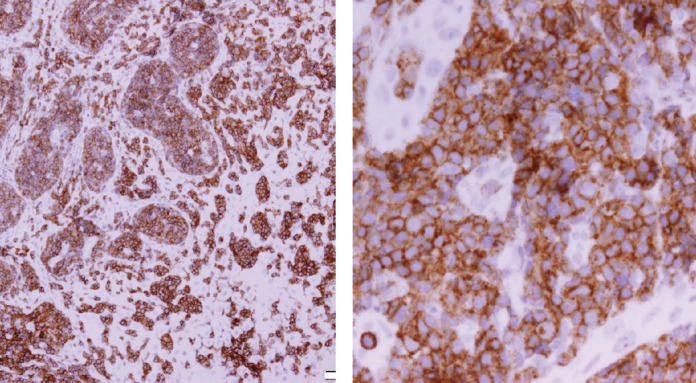
Immune-histochemical staining of the same sections showing CD4-positive cells (×20: left side, ×40: right side).

**Figure 9.**
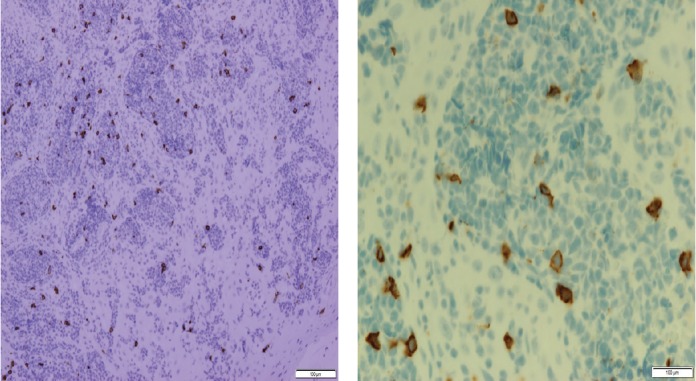
Immune-histochemical staining of the same sections showing CD8-negative cells (x20: left side, x40: right side).

Histopathologic examination of pharyngeal biopsy revealed infiltration by malignant lymphoid cells with hyperchromatic enlarged nuclei and scanty cytoplasm (Figures 5 and 6) while immune-histochemical staining showed CD3 (+), CD4 (+) and CD8 (–) cells (Figures 7–9). A picture compatible with ATL. On the other hand; duodenal biopsy showed non-specific inflammatory changes with lymphocyte and plasma cell infiltration.

### Treatment

As laboratory investigations, imaging findings and tissue diagnosis were consistent with the aggressive subtype of smouldering ATL (leukaemic subtype), intensified chemotherapy was started using mLSG-15 protocol (VCAP-AMP-VECP) in addition to intrathecal cytarabine, methotrexate and prednisone. The patient partially improved following chemotherapy and will be scheduled for stem cell therapy.

## Discussion

The 4th Japanese nationwide survey of 657 patients with all subtypes of ATL from 1986 to 1987 demonstrated that 5% of ATL patients were from Kyushu Island and the average age of patients was 57.5 for males and 57.8 years for females [10].

Oral manifestations of lymphoma are often difficult to be diagnosed because it seems to occur very rarely with few cases being reported in the medical and dental literatures. Also, it usually presents with clinical features that mimic other diseases such as periodontal disease, osteomyelitis and other malignancies which may delay the correct treatment thereby worsening the prognosis [11].

Our patient was a 49-year-old female, living in an HTLV-1 endemic area (Nagasaki prefecture, Kyushu Island, Japan). Initially, she presented with ATL lesions in the skin and oral mucosa which were misdiagnosed as fungal infection. Routine laboratory investigations revealed atypical lymphocytosis with normal LDH, normal serum calcium and negative ANA, which helped to exclude an auto-immune disorder like systemic lupus and directed attention towards lymphoproliferative disorders. HTLV-1 infection was confirmed in our patient by detection of monoclonal bands using the Southern blot technique.

Screening upper endoscopy and NBI provided a clue towards diagnosis by detection of thickened aryepiglottic folds suspicious of infiltration and multiple characteristic ulcers in the palate, gingiva, uvula and pharynx (Figures 1–3) which were biopsied. CT and PET-CT findings showed disseminated disease affecting skin, lymph nodes and lungs, with special predilection to oropharyngeal mucosa demonstrated by dense uptake of FDG in the pharynx, palate and tip of the tongue. The final diagnosis of our case was based on histopatholgic examination and immune-histochemical staining of the endoscopic specimens which revealed a picture compatible with smouldering type ATL (Figures 5–9).

The Japan Clinical Oncology Group revealed that aggressive ATL has a very poor prognosis compared with other types of aggressive non-Hodgkin lymphomas and the best chemotherapy for patients with aggressive ATL should be dose-intensified multi-agent chemotherapy including VCAP-AMP-VECP plus intrathecal cytarabine, methotrexate and prednisolone [12, 13]. However; allogeneic hematopoietic stem cell transplantation inducing graft-versus-ATL effect following myeloablative or reduced intensity conditioning regimens has been reported to cure some patients with aggressive ATL despite its high transplant-related mortality [14, 15]. As our patient did not achieve full improvement following intensified chemotherapy (mLSG-15) protocol, she was scheduled for allogenic stem cell therapy.

Many previous studies have reported atypical presentations of T-cell leukaemia/lymphoma such as variable oral lesions [11] or cutaneous manifestations [16] in patients with ATL, renal infarction subsequent to polyarteritis nodosa in a patient with angioimmunoblastic T-Cell lymphoma [17], and testicular and uterine mass lesions in two patients with T-cell lymphoblastic lymphoma [18, 19].

## Conclusion

In this case report, we recorded unusual presentation of ATL and demonstrated its therapeutic challenge for raising awareness of endoscopists, physicians and dentists about this disease especially in HTLV-1 endemic areas to avoid late diagnosis and aid proper therapeutic decisions.

## Conflict of interest

Authors declare there is no conflict of interest related to this case report.

## Figures and Tables

**Table 1.** Blood indices of the patient with its reference ranges.

Blood indices	Patient’s result	Reference range
Red blood cells	4.4 × 10^6^	3.8–4.9 × 10^6^
Haemoglobin	13.4	11.6–14.8 g/dL
Platelets	274	158–348 × 10^3^
Leucocytes	8.3 × 10^3^	3.3–8.6 × 10^3^
Blast	0%	0%
Pro-myelocyte	0%	0%
Myelocyte	0%	0%
Meta-myelocyte	0%	0%
Stab	1%	0.5–6.5%
Segmented	45%	38–74%
Lymphocyte	10% (Low)	16.5–49.5%
Monocyte	0 (Low)	2–10%
Eosinophil	3	0–8.5%
Basophil	0	0–2.5%
Atypical lymphocytes	1 (High)	0%
Abnormal lymphocytes	40 (High)	0%
Others	0%	0%

**Table 2.** Important biochemical and immunological investigations of the patient with their reference ranges.

Biochemical investigations	Patient’s result	Reference range
Ca	9.3	8.8–10.1 mg/dl
Na	140	138–145 mmol/L
K	3.9	3.6–4.8 mmol/L
Cl	102	101–108 mmol/L
BUN	9	8–20 mg/dL
Creatinine	0.6	0.4–0.7 mg/dL
Uric acid	3.5	2.6–5.5 mg/dL
Total protein	7.7	6.6–8.1 g/dL
Albumin	4.8	4.1–5.1 g/dL
Total bilirubin	0.5	0.4–1.5 mg/dL
AST	11	13–30 U/L
ALT	9	7–23 U/L
LDH	172	124–222 U/L
CK	49	41–153 U/L
Glucose	107	73–109 mg/dL
eGFR	82	70–110
CRP	0.04	0–0.1 mg/dL
ANA	-ve	<10 IU/mL
RF	4.7	<15 IU/mL
EBV	-ve	-ve
HTLV	>45 (High)	<1 Cutoff Index (COI)
s IL-2R	3420 (High)	<800 U/mL in HTLV-1 Asymptomatic carriers


ALT: Alanine transferase, ANA: antinuclear antibody, AST: aspartate transferase, BUN: blood urea nitrogen, CK: creatine kinase, EBV: epstein barr virus antibody, HTLV: human T-lymphotropic virus-1 monoclonal bands, RF: rheumatoid factor, eGFR: estimated glomerular filtration rate, sIL-2R: soluble interleukin-2 receptors
